# The Hormesis Concept: Strengths and Shortcomings

**DOI:** 10.3390/biom13101512

**Published:** 2023-10-12

**Authors:** Stephen C. Bondy

**Affiliations:** 1 Center for Occupational and Environmental Health, Department of Environmental & Occupational Health, University of California, Irvine, CA 92697, USA; scbondy@uci.edu; 2 Department of Medicine, University of California, Irvine, CA 92697, USA

**Keywords:** hormesis, dose response, biphasic, toxicity, radiation, exposure

## Abstract

Hormesis implies that the effects of various materials or conditions that organisms are exposed to, may not have linear dose-response characteristics but rather, can be biphasic. Thus the response to a low dose of a stressor may be the opposite to that occurring at higher doses. Such a dual response is postulated for many toxicants and physical conditions and may involve a beneficial adaptive response. Such a non-linear effect is undoubtedly present in many useful pharmacological and nutraceutical agents with can be toxic at high concentrations. This somewhat divisive topic is an area of study that should be objectively studied and not clouded by political and policy considerations. The objective of this review is to examine claims concerning those exposures where hormesis seems to exist and also those where there is no good supporting evidence. The breadth of this phenomenon and potential mechanisms underlying hormetic events are discussed together with their limitations.

## 1. Introduction

The concept of hormesis reflects the finding that many agents and environmental conditions can have opposing effects at low or high doses. Small amounts of a stressor or toxin may provide protection against subsequent higher doses of the harmful agent in question or against the damage caused by a different adverse event. This is known as the “adaptive response” or “pre-conditioning”. Some form of this kind of protection has been known for millennia. King Mithridates VI of Pontus (135–63 B.C.), a temporarily successful opponent of Roman rule, was a keen student of pharmacology and protected himself from poisoning by ingestion small amounts of poisons intended to develop resistance to toxic doses of various poison. In this way he escaped the fate of his father who died by poison. More formally, Schultz [1] first reported a biphasic dose response of the viability of yeast exposed to varying amounts of disinfectants.

Nearly all desirable foodstuffs, vitamins, nutritional supplements and water can be harmful if consumed in excess. Pharmacological agents have an effective dose range and if this is exceeded, toxicity is generally the result. The therapeutic index relates the amount of a drug that is therapeutically effective to that which causes toxicity. The margin of safety between clinically effective dose and a toxic dose can vary considerably. The narrow therapeutic index in the case of opiates, accounts for the high mortality found among users of this drug [2]. One the other hand antacids have a very high toxic threshold. Many vitamins have similarly high toxic thresholds and thus good margins of safety. Environmental conditions such as ambient temperature all have a desirable mean that supports life effectively, and can be harmful if deviating to much from this. The commonality of biphasic responses of organisms to chemicals or environmental changes is indisputable [3]. Low levels of adversity often lead to metabolic adjustment while higher exposures can be lethal to a cell. Vaccination and immunization involve the initial administration of a non-infectious material that contains the immune-provoking component of a bacterium or virus in order to minimize the effect of subsequent infection by such organisms. Since in this case the original stimulus differs from the agent it is conferring protection from, it cannot strictly speaking be considered as a hormetic effect. However, such clinical procedures are un-doubtably based on the hormetic concept of a tolerable stimulatory challenge giving sub-sequent protection to a more severe subsequent exposure to a similar pathogen. The initial response to a low level stimulus may reduce the magnitude of the response to later, higher levels of an adverse stimulus and thus act in a protective manner. These features are unsurprising and almost any successful organismic adaptation to an exogenous stimulus can be described as hormesis. Thus, there is a danger that the term hormesis can be used so broadly as to lose any useful and concise meaning. This survey attempts to focus on aspects of hormesis other than those that are rather self-evident.

**Figure 1:**
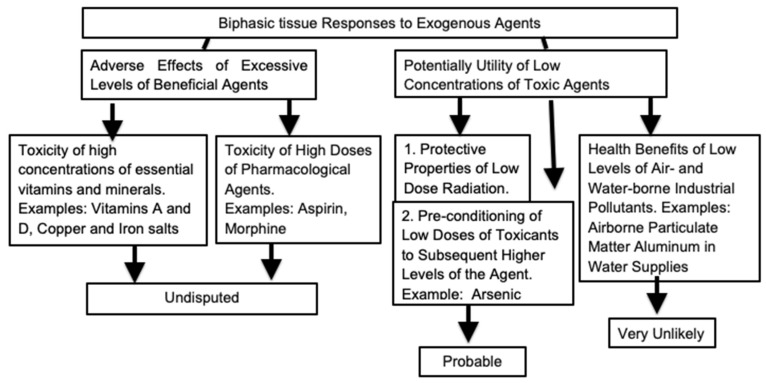
Classes of phenomena described as hormetic responses.

This report initially discusses the biphasic responses within cells, either to intrinsic constituents or to nutritional and lifestyle factors. It then proceeds with evaluation of the effects of low levels of radioactivity. Potential hormetic responses following exposure to such radiation has been widely reported. Radiation is the most studied exogenous factor that is undoubtedly harmful at high levels but which may have protective properties at lower levels. The final section concerns the relevance of hormetic events that are likely to have positive qualities, to chronic exposures to industrial pollutants. This extrapolation has been used in order to justify several types of low-level environmental contamination. The hazards of using hormetic phenomena in order to validate atmospheric or water-borne pollution are described. The directions followed by this overview are summarized in Figure 1.

## 2. Problems with Evaluating the Current Literature

The topic of hormesis a sufficiently controversial so as to have generated severe criticism and passionate support. Both of the attitudes tend to make objective interpretation of some of the literature, difficult. The issue underlying these opposing views, relates to the harmfulness of low levels of environmental contaminants, know to be toxic at higher levels. A lot is at stake in deciding whether toxicants can actually be beneficial at low enough concentrations which could be used to justify acceptance of elevated levels of industrial effluents in the environment. This has led to the insertion of significant political input into the debate, which by its very nature cannot be considered unbiased. This review has attempted to mitigate this by uncovering literature from a wide range of sources. A few scientists have published overwhelmingly in this field, repeatedly promoting their perspective. Some of these have received financial support from polluting industries. Whether these approaches can be considered impartial has been questioned [4,5]. In order to maximize diversity of opinion we have attempted to reduce such dominance by more prolific authors by using reports from the largest possible range of sources and whose conclusions are not obviously aligned with interests of sponsors. We include key findings in this somewhat contentious area in as unbiased a manner as possible.

Another difficulty in evaluation of hormesis-related literature is that it can sometimes be used in justification of, and doorway leading to dubious concepts such as homeopathy [6,7,8,9]. This view of science constitutes a departure from modern allopathic medicine. Homeopathy claims a utility of ultra-low levels of very toxic chemicals such as mercury salts (*mercurius solubilis*) for a wide range of health conditions. This connection between homeopathy and hormesis has been used to validate the assertion that low levels of highly toxic and persistent dioxins may have potentially healthful effects [10] but such reports are described as “alarming and contradicted by a large literature from the field of endocrine disruption” by more orthodox scientists [11]. Making such a connection between hormesis and homeopathy for mutual substantiation, can be perilous [12].

The term hormesis is commonly misused in order to describe events found only at low concentrations [13]. For example, it is not accurate to describe the ability of *N*-acetyl cysteine to moderate exercise-induced oxidative stress as a hormetic event [14].

## 3. Intrinsic Events

Several types of environmental exposure, when present early in development or at low concentrations, have been shown to confer beneficial effects while at higher doses, they are recognized as toxic. These include events such as intermittent hypoxia, responses to low levels of inflammation-stimulating components of biological toxins. Early exposure to allergens can give protection later in life.

Intrinsic biochemicals such as nitric oxide, β-amyloid peptide, inflammatory cytokines, the neurotransmitter glutamate and various hormones also trigger biphasic responses with the lower levels being beneficial [15,16]. The electron transport chain within aerobic mitochondria links the oxidation of chemicals progressively and sequentially, to the formation of a proton gradient across the inner mitochondrial membrane. The dispersal of this gradient then effects the production of ATP. While this is generally a very efficient process, as minor leakage of unstable oxidant intermediates occurs. This is the source of much of the reactive oxygen species (ROS) that can be damaging to macromolecules within the cell. Many intracellular defenses exist that mitigate this hazard. One significant response that can be taken as hormetic is the induction of vital signaling pathways by low levels of ROS. These transcription pathways then activate the antioxidant response element (ARE) which regulates the transcription of a suite of genes for antioxidant enzymes. By this means the cell is better prepared to resist higher levels of ROS. Low levels of ROS are involved in optimization of cell signaling while higher levels can have adverse sequelae [17]. The induction of this protective response forms the means of action of many agents and various physiological states some of which are briefly mentioned below.

## 4. Health Value of Moderate Exposure to Physical and Dietary Stressors

The development of tolerance following mild or intermittent exposure to conditions that would be harmful if excessive or continuous is well recognized [18]. These often pertain to lifestyle factors. Quantitative and temporal aspects of diet including calorie restriction and intermittent fasting are also thought to be challenges that prime the metabolism to withstand greater stresses [19]. The likely health effects of transient hyperthermia due to sauna use may also function in this manner [20]. Similarly, transient or low level exposure to exercise can also act as as useful preparation for later more extreme challenges [21,22]. There can be overlap between harmful and beneficial effects. The ROS produced by excessive exercise, can be harmful to muscle tissue and simultaneously induce activation of protective signaling pathways, thereby leading to adaptive changes in the muscle [23].

There is good evidence supporting the importance of intermittent manageable challenges in slowing the rate of senescence. Beneficial low level stressors include a diet rich in fruits and vegetables diet containing a panoply of phytochemicals. Many of these are minor stressors and provoke the induction of cellular defense mechanisms. Activation of the Nrf2-ARE signaling pathway, and of DNA repair processes are examples of such effects [24]. Increasing the alertness posture of an organism in this manner may retard age-related changes while higher levels of similar agents and physical conditions can result in acceleration of the progression of adverse changes and consequent increased rate of aging [25]. It has been proposed that one of the benefits caused by dietary induction of ketosis (as a means of inducing weight loss) is the promotion of minor oxidative stress which can lead to induction of nuclear factor erythroid 2-related factor 2 (Nrf2), and sirtuins and thence to consequent elevated presence of anti-oxidant and anti-inflammatory factors [26]. Most age-related diseases are due to the organism’s excessive and prolonged response to the primary initiating injury rather than directly to the original causal stimulus. This leads to a chronic condition where inflammatory and pro-oxidant events become continuous and independent of the initiating pathological event. Such continuing overreaction seems to characterize several major neurodegenerative disorders such and Alzheimer’s and Parkinson’s disease [27]. The triggering element can be of exogenous origin (e.g., airborne particulate matter) or intrinsic (e.g., amyloid peptide deposition). The prior induction of protective anti-oxidant and anti-inflammatory pathways by parallel stressors at controlled levels can effectively mitigate the later effects of more intense challenges [28]. These biphasic effects on cellular health are hormetic in nature. However, the balance between desirable and harmful effects of apparently minor stressors remains complex and outcome can be uncertain [29].

## 5. Dietary Components with Biphasic Effects on Wellbeing

A wide range of vitamins and phytochemicals that have been reported as beneficial at low concentrations may be harmful at higher levels. Such phytochemicals include curcumin, resveratrol, tomatidine and Scutellaria [30,31,32]. Most of the anti-oxidative mechanisms effected by phytochemical involve secondary events, notably changes at the level of gene expression due to induction of key transcriptional and trophic factors. Unlike direct antioxidants effects such as enabled by some vitamins, responses to phytochemicals are more subtle and likely to be hormetic in that they may involve transient elevation of mitochondrial ROS production [33]. The activation of stress response pathways in this manner may underlie the utility of the Mediterranean diet (which is rich in fruits and vegetables) in slowing the progression of events associated with senescence [34]. Another crucial difference, in comparison with direct antioxidants, such an indirect metabolic activation could result in extension of the period of efficacy of phytochemical action. A caveat concerning many nutraceutical reports is that they have the limitation that to be often based solely on in vitro studies where the extent of intestinal degradation and absorption cannot be considered [13,35].

## 6. Radiation and Hormesis

Perhaps the most widely studied and the most contested topic in the field of hormesis is the possibility that low levels of radiation may produce healthful changes. Despite many reports and analyses, the issue has not been fully clarified. This is in part due to the difficulty of working at the lower end of the dose-response curve in detecting significant deviation from a linear dose-effect relationship. Many data points are needed when to allow detection of a postulated U-shaped response curve. In epidemiological studies, use of such large numbers in search of a minor effect, increases the possibility of findings being clouded by confounding factors. In consequence, since the issue remains unsettled, the application of risk-benefit logic by regulatory agencies has generally been to dismiss the possibility of radiation being desirable at any level. Nevertheless, the scientific method demands analysis of the issue without any social or political considerations. There are in fact many varied reports suggesting that low levels of ionizing radiation may have positive effects on health. These are most commonly of epidemiological origin but results from clinical research, animal models and cell culture are also to be found.

### 6.1. Epidemiological Reports

Many of these are derived from inadvertent exposure to radioactive materials resulting from the use of the atom bomb, in warfare and in testing. It is recognized that high exposures increase the incidence of many forms of cancer, notably leukemia. At such levels, the dose-response relationship between radon and lung cancer mortality is linear [36]. Similarly, the incidence of lung cancer related to residential exposure to radon is elevated in proportion to radiation dose levels over 100 Bq m^−3^ [37]. However, there are many reports that the dose-effect relationship breaks down at lower levels of radiation. Not only does radiation due to radon at a certain concentration fail to increase incidence of lung cancer, but it seems to exert a protective effects [38,39]. Protective effects of low dose radiation on lung cancer have also been reported in areas close to sites of testing of nuclear weapons [40]. There are several descriptions from both animal models and epidemiological studies on low levels of radiation producing protective hormetic effects [41]. However, in view of the difficult problem of analyzing the effects of low dose radiation, more recently such findings have been frequently contradicted [42]. These conflicting reports illustrate the difficulty of analyzing and arriving at unambiguous conclusions at the lower end of dose response curves. An inverse correlation has been found between levels of natural background radiation and the mortality rate in a regional analysis of the United States [43]. The number of potential confounders in such large epidemiological surveys is manifold. A meta-analysis of the data relating to the credibility of the linear no-threshold model of toxic exposure, concluded that while this idea is not conclusively disproven, the breadth and variety of these reports has led to the need for reconsideration of this paradigm, rather than considering it unquestionably established [44]. It has also been proposed that there is sufficient critical evidence that would justify raising acceptable exposure limits [45].

### 6.2. Clinical Reports

While radiation is a standard treatment for many forms of cancer, the utility of radon in this context, only its use in treatment of non-malignant disease is addressed here. Radon is widely used as a treatment for arthritis and this may be due to inhibition of inflammatory events [46]. Exposure to radon mitigates asthma in humans [47] and this has been confirmed using an animal model [48]. The utility of this strategy on other inflammation-related disorders such as Alzheimer’s disease is currently being investigated [49]. Some utility of radiation has been found to limit acute inflammatory pulmonary events in COVID-19 [50]. Although radiation seems to suppress excessive inflammatory responses, it may promote desirable immune activity [51]. In this it resembles the similar dual effects of several phytochemicals.

Inadvertent low dose of radiation scatter received by the ovary, following radiation treatment of rectal or rectosigmoid junction cancer, led to a significant reduction of ovarian tumor levels relative to an untreated population, clearly a hormetic effect [52].

### 6.3. Findings from Isolated Systems and Animal Models

Such reports are critical in order to validate epidemiological research. Furthermore, this laboratory-based direction can lead to improved mechanistic understanding and the possibility of establishing radiation hormesis on more widely accepted and firmer footing, is increased.

The use of isolated systems indicates that the type of radiation used can influence the nature of resulting damage and that mitochondria are likely to play an important role in any kind of hormetic effect [53].

Fisher and Weller (2010) have summarized an extensive series of lifespan studies performed at the Pacific Northwest National Laboratory, on the effect of irradiation of dogs by inhalation of radioactive plutonium [54]. A protective effect of low dose exposures was found in that the incidence of lung tumors in untreated control dogs was significantly greater than the incidence in dogs exposed to low dose plutonium. Additionally, there are data suggesting that prior exposure to low dose irradiation (10cGy 48 h) can significantly increase the survival rate of mice with bacterially induced systemic sepsis. This was associated with induction of Nrf2, reduction of cytokine levels and NO and reduction of the bacterial burden [55].

### 6.4. Closing Comment

Animal models directly addressing hormesis are scarce but valuable, as conditions of irradiation and homogeneity of population can be much better controlled than in human studies. Most studies have involved invertebrate rather than mammalian models [45]. While there are several relevant articles from the last century utilizing mammalian species, the area seems to have fallen into abeyance and there are few more recent reports. More such animal studies would help to consolidate the radiation hormesis concept [56]. Despite extensive studies over many years, many key questions concerning the relation of dose rate in effecting radiation-induced tissue damage, remain unanswered. However, overall evidence suggests that very low dose rates have often been found to increase lifespan through a mechanism likely to involve adaptive responses. An inverse dose rate response has been found in studies performed below levels of natural background radiation [57]. While the authors of this recent review do not use the word, this effect is undoubtedly hormetic.

Overall, the uncertainty of establishing the extent and value of hormetic events after exposure to radiation due to the difficulty of working at the lowest levels of a dose-effect curve has resulted in excessive caution on the one hand [58], and excessive passion on the other [59] where words like “radiophobia” are used to mock concerns about safe levels. Such levels are accused of being set for economic and political reasons rather than being determined scientifically [60]. Several complex statistical approaches involving evaluation of probabilistic risk factors and linear non-threshold theory, have been used to validate either opinion or to suggest ways forward [61,62].

## 7. Conclusions

The underlying supporting basis for these diverse studies, is that a minor stressor can pre-condition an organism to more severe subsequent exposure to the same stressor [63]. Activation of immune responses by pre-conditioning by using similar materials, is likely to be a major means of inducing tolerance to pathogens or toxicants. This is a very reasonable and widely accepted concept, as exemplified by the success of vaccination. There is much mechanistic evidence that can illuminate the basis for the existence of hormetic events in biology. Many realistic potential underlying modes of action of this phenomenon exist. Living organisms have an intrinsic drive to respond to any alterations in the surrounding milieu by making appropriate metabolically organized adjustments designed to maintain homeostasis exist. This attempt to maintain equilibrium is sustained until stressors become excessive, after which the breakdown of ability to protect the internal milieu of the cell, leads to its injury or death. The molecular events that mediate such responses within the cell, take place by enhanced production of anti-oxidant and anti-inflammatory proteins due to the presence of modest elevation of a challenging exogenous environment. This includes stimulation of pathways leading to production of antioxidant and anti-inflammatory protective proteins thus promoting recuperative events. These preparatory pathways are enabled by activation and nuclear transfer of transcription factors such as Nrf2 and NF-κB, leading to the genetic derepression of suites of protective genes, such as the antioxidant response element (ARE) [64]. A noteworthy outcome of the presence of a pathway common to various types of induced stress is that there may be protective effects of an initial specific challenge upon other subsequent unrelated types of cellular stress [15]. Such adaptive preconditioning can account for the biphasic effect of different levels of a chemical or an environmental condition, upon an organism, and this forms the basis of hormesis.

Much evidence is generally supportive of possible hormetic events attributable to natural materials and conditions which organisms are likely to have genetic familiarity over many generations. This probably applies to phytochemicals, radiation and climactic conditions such as temperature. However, it is an unjustified step to then assume that parallels are to be found in the vast area of man-made industrial pollutants with which organisms do not have an evolutionary history of prior experience. This is also likely to be the case of toxic metals whose concentration was originally a very small fraction of current levels. For example the global atmospheric content of mercury has increased enormously in the last 600 years, as judged by the analysis of an ice core from Mount Logan, Yukon, Canada [65].

Hormesis has been described as “becoming prominent in leading textbooks in toxicology, pharmacology, and related biomedical areas” [66]. However, this is not borne out by the report that the number of hormesis publications per year over the last decade has seen a slow decline which has been interpreted as “suggesting that hormesis is becoming less important in toxicological research, and that toxicologists have deemed that there is not enough evidence to support the concept being an important part of chemical risk assessment” [67]. These contradictory positions reflect the difficulty of impartial evaluation of current information. This may in part be due to the unquestioned importance of this area of study. The legislation of Public Health decisions concerning what should be acceptable levels of environmental contamination by various pollutants is critical. Hormesis means that some individuals may benefit from low exposure to toxicants. However, this may not apply universally to an entire heterogeneous population which includes immature infants with markedly different susceptibilities than those of adults [68]. Consequently, toxic chemical at a given level may be beneficial to one individual and harmful to another.

At the molecular level of a single organism, it is likely that amounts of a toxicant inducing beneficial defensive responses at one site, can simultaneously cause adverse effects in other areas. Such overlap of desirable and harmful effects, combined with the diversity of the human population suggests that great caution must be used in advocating changes to the linear dose response model, despite that it is unlikely to be totally accurate. In addition to the general population, there are many powerful stakeholders with significant economic interests vested in the outcome of the legislative process concerning setting of acceptable lows of harmful materials. In view of the diversity of human susceptibility and variance in levels of potential exposure, it has been been stated that the danger of incorporating hormetic concepts to such decision making, could be hazardous [69,70,71]. Furthermore, it should be taken into account that the positive events at the lower end of a biphasic dose-response curve are much more circumscribed and limited than the potentially adverse effects at the upper end of the curve which can be extended indefinitely.

Nevertheless, it is important that the significance of the topic be objectively evaluated leaving aside all sociopolitical and economic aspects. There is no question that many effects of beneficial materials can be harmful at excess levels. The more contentious questions concern the potential benefits of low concentrations of toxic materials of anthropogenic origin where commercial interests can be severely impacted by regulatory decisions.

Since the term hormesis is sometimes used passionately as an almost faith-based universal truth underlying most biological phenomena, it might be better to use narrower and more precise terms in describing specific phenomena. Explicit phrases such as ‘non-monotonic dose response curves’, ‘biphasic responses’, ‘adaptive reactions’ ‘inverse dose-effect’ or ‘pre-conditioning’ which are more concise and focused, may be easier to accept. This could serve to encourage detachment and lead to more constructive discussions.
